# Canadian COVID-19 Crisis Communication on Twitter: Mixed Methods Research Examining Tweets from Government, Politicians, and Public Health for Crisis Communication Guiding Principles and Tweet Engagement

**DOI:** 10.3390/ijerph19116954

**Published:** 2022-06-06

**Authors:** Melissa MacKay, Andrea Cimino, Samira Yousefinaghani, Jennifer E. McWhirter, Rozita Dara, Andrew Papadopoulos

**Affiliations:** 1 Department of Population Medicine, University of Guelph, Guelph, ON N1G 2W1, Canada; ciminoa@uoguelph.ca (A.C.); j.mcwhirter@uoguelph.ca (J.E.M.); apapadop@uoguelph.ca (A.P.); 2 School of Computer Science, University of Guelph, Guelph, ON N1G 2W1, Canada; syousefi@uoguelph.ca (S.Y.); drozita@uoguelph.ca (R.D.)

**Keywords:** COVID-19, social media, Twitter, crisis communication, social media engagement

## Abstract

To foster trust on social media during a crisis, messages should implement key guiding principles, including call to action, clarity, conversational tone, compassion and empathy, correction of misinformation, and transparency. This study describes how crisis actors used guiding principles in COVID-19 tweets, and how the use of these guiding principles relates to tweet engagement. Original, English language tweets from 10 federal level government, politician, and public health Twitter accounts were collected between 11 March 2020 and 25 January 2021 (*n* = 6053). A 60% random sample was taken (*n* = 3633), and the tweets were analyzed for guiding principles. A tweet engagement score was calculated for each tweet and logistic regression analyses were conducted to model the relationship between guiding principles and tweet engagement. Overall, the use of guiding principles was low and inconsistent. Tweets that were written with compassion and empathy, or conversational tone were associated with greater odds of having higher tweet engagement. Across all guiding principles, tweets from politicians and public health were associated with greater odds of having higher tweet engagement. Using a combination of guiding principles was associated with greater odds of having higher tweet engagement. Crisis actors should consistently use relevant guiding principles in crisis communication messages to improve message engagement.

## 1. Introduction

The COVID-19 pandemic changed Canadian life as various provinces declared multiple states-of-emergency, non-essential businesses and schools closed, and public gatherings were restricted. Information from public health and decision-makers were essential to inform the population of these restrictions and highlight the public’s role in diminishing the negative impact of COVID-19 within their communities. The content, timing, and chosen communication channels for disseminating information are vital to addressing uncertainty surrounding an emerging infectious disease such as COVID-19 and minimizing the burden of disease [1].

To ensure a systematic and comprehensive approach to risk and crisis communication, guidelines, reports, and research have focused on identifying guiding principles or best practices that are audience-centered, incorporate the processes of risk perception, and take into account the structural and contextual factors that are associated with the emerging infectious disease [2,3,4,5,6,7]. The use of guiding principles for effective crisis communication ensures messages are clear, targeted and tailored, timely, and foster trust among the public, which impacts acceptance of messages and uptake of public health recommendations [3,7,8,9]. Importantly, transparent messages help the public make sense of any uncertainty surrounding an emerging infectious disease and disclose how official crisis actors (e.g., government officials, politicians, and public health officials) are responding to the crisis [2,10]. Guiding principles provide balanced information that meets the needs, wants, and interests of various segments of the public, which helps to cut through mis- and dis-information [2,11]. The use of guiding principles balances the information needs of individuals with information that influences risk perception and behaviors that are necessary to minimize morbidity and mortality that are associated with an emerging infectious disease [3,9,12].

Risk perception and risk protective behaviors are highly influenced through the effective use of social media [13]. Social media has allowed the public to move from only being consumers of crisis messages to being able to create, respond to, and share information as well [14]. The inherently interactive and immersive aspects of social media facilitate user information sharing, co-production, and participation [15]. The interactive nature is an important outcome of strategic communication that contributes to message acceptance, trust, and uptake of recommendations [15]. Social media platforms have technological attributes that enable interaction such as sharing, likes or other reactions, and comments or replies. Metrics that are related to these interactive attributes can indicate an individual’s opinion on the content [16] and influences others to similarly evaluate the information and source [17].

Twitter, a micro-blogging site, is one of the most popular social media platforms in Canada, with over 6.45 million active users [18]. Weekly Twitter use in 2019 was similar across age groups with 30% of users between 23 and 28 years old, 33% between 39 and 54 years old, and 27% over 55 years old [19]. Among the interactive features of Twitter, retweets and replies have been found to impact attitudes and judgements towards the message and source [20]. Such metrics can function as a bandwagon cue, where an individual uses them to assess if others accept the message [20]. Social media monitoring provides an important tool for crisis communication actors to assess the public’s acceptance of messages and how they are coping with threats within crisis rhetorical subarenas such as Twitter [14,21]. Twitter has specifically been identified as an important listening tool for evaluating the public’s needs and concerns regarding a crisis [21].

Effective crisis communication on social media involves including relevant guiding principles in messages and maximizing the social function of platforms and monitoring the public’s response to messages. Key guiding principles on social media for effective crisis communication to maintain trust during emerging infectious diseases include call to action, clarity, compassion and empathy, conversational tone, correction of misinformation, and transparency (Table 1) [4]. Table 1 was developed from a literature review examining the best practices and evidence surrounding crisis and health communication during emerging infectious diseases, including through the use of social media [4].

Research examining tweets by federal actors during COVID-19 in Canada have focused on message function such as use of hashtags and the relationship to engagement [28], and message form such as providing information about COVID-19 risk [29]. No research has focused on the guiding principles for effective crisis communication using Twitter in Canada. The goal of this research is to evaluate the quality of Canadian government, politician, and public health crisis communication on Twitter related to COVID-19, and how the quality of their tweets (i.e., use of guiding principles) impacts public engagement. With this study we aimed to:

1. Describe how included actors are incorporating guiding principles for effective crisis communication in COVID-19 related tweets;
2. Evaluate the relationship among guiding principles, sources, and tweet engagement; and,
3. Evaluate the relationship between the number of guiding principles that are used per tweet and tweet engagement.

## 2. Materials and Methods

### 2.1. Data Collection

Tweets that were posted by 10 Canadian government, politician, and public health Twitter accounts that were relevant to the national level (Table 2), as well as engagement metrics (i.e., number of retweets, number of tweet favorites, number of tweet quotes, and number of tweet replies), were collected. Government accounts included all organizations that were related to federal level health that tweeted about COVID-19 before data collection. Politician accounts included federal leaders of official parties in Canada, with the exception of the leader of the Bloc Quebecois as the tweets did not meet the English language inclusion criteria. Public health accounts includedfederal-level organizations and leaders that were responsible for the programs, policies, and services that promote and protect the public health of Canadians. The Twitter Premium Search API was used with keywords including COVID, COVID-19, coronavirus, and SARSCoV2 to retrieve COVID-19-related tweets from the aforementioned accounts. Original, English language tweets (i.e., no retweets or reply tweets from accounts and third-party applications) that were posted from 11 March 2020 to 25 January 2021 were included for collection.

Tweets were organized according to the source of the tweet (i.e., by Twitter account name) and subsequently classified into the following three categories: government, politician, and public health Twitter accounts.

### 2.2. Content Analysis

A total of six guiding principles for effective crisis communication to maintain trust [4], were used to assess the quality of crisis communication of tweets that were included in this study. The guiding principles for crisis communication were developed from a review of the literature including grey literature such as the Centers for Disease Control and Prevention’s Crisis and Emergency Risk Communication manual, combining best practices for crisis communication and social media communication [4]. Table 1 describes the six guiding principles that were used and includes an example tweet for each.

A codebook describing each guiding principle was created, and a codebook training session with the involved researchers took place before coding began. Two researchers each independently coded a random sample of the data (*n* = 151) for the presence or absence of each guiding principle [30]. A kappa score of 0.86 was obtained when coding of the random sample was compared between the researchers, indicating high reliability [31]. All conflicts were discussed and resolved before a 60% random sample of the data was taken (i.e., every 3 of 5 tweets selected) and then split equally among the two researchers to complete coding.

### 2.3. Statistical Analysis

Basic descriptive analyses were performed using Excel version 16.50 (Microsoft Corportation, 2018, Albuquerque, MA, USA) to assess how each of the guiding principles were used. To model the relationship between tweet engagement rate and guiding principles, a series of logistic regression analyses were conducted. Logistic regressions were used to understand if the expected outcome significantly differed from the observed outcome. The AUC (area under the curve) was used to see how well our model distinguished between average engagement and low engagement. Tweet engagement rate was calculated for each tweet by adding the number of replies and retweets, then dividing this sum by the number of followers of the account tweeting [32]. Tweets with an engagement rate of 0.5 or more were classified as having an average engagement, while tweets with an engagement rate of less than 0.5 were classified as having a low engagement [32]. First, we conducted univariate logistic regressions to determine how the presence of each guiding principle affected tweet engagement. We then added in tweet source (i.e., government, politician, or public health account) as a predictor variable and conducted multivariate logistic regressions to see how the source and the presence of each guiding principle affected tweet engagement. Lastly, we conducted univariate logistic regression models to assess how the number of guiding principles that were used in a tweet affects tweet engagement. All logistic regression models were conducted in RStudio version 1.4.1717 (RStudio Team, 2015, Boston, MA, USA).

## 3. Results

A total of 6053 original tweets were collected after retweets and replies were removed. The 60% random sample left 3633 tweets to be analyzed.

### 3.1. Use of Guiding Principles

The use of guiding principles varied greatly across Twitter accounts and each individual guiding principle (Table 3). Call to action was the most widely used guiding principle, used in 97.08% of government tweets, 87.77% of politician tweets, and 65.87% of public health tweets. Conversational Tone followed as the second most widely used guiding principle, used in 30.84% of government tweets, 61.47% of politician tweets, and 50.88% of public health tweets. Of particular note, Prime Minister Justin Trudeau used conversational tone in 95.59% of his tweets, and Canada (general Canadian governmental organization account), Deputy Prime Minister Chrystia Freeland, and Health Canada and Public Health Agency of Canada (PHAC) used it in over 60% of their tweets. The least used guiding principle was correction of misinformation, with most accounts not using it in any of their tweets. Clarity, compassion and empathy, and transparency were also used infrequently. Clarity was used in less than 17% of tweets from all accounts except for Canada (45.45% of tweets) and Health Canada and PHAC (49.59% of tweets). Compassion and empathy were used mostly by Chrystia Freeland (61.54% of tweets), but only used in 3% to 33% of tweets from all other accounts. Similarly, transparency was used by Canada in 36.36% of their tweets and the Canadian Institutes of Health Research (CIHR) in 19.82% of their tweets, but in 0% to 5% of tweets from all other accounts.

### 3.2. Relationship between Guiding Principles and the Level of Tweet Engagement

Table 4 shows results from the univariate logistic regression models assessing how each guiding principle affects tweet engagement. When compassion and empathy is present, the odds of receiving an average rate of engagement were 2.2 times greater than when compassion and empathy is absent (ß = 0.770; *p* = <0.001). Similarly, when tweets were written with a conversational tone, the odds of receiving an average rate of engagement were 2.8 times greater than when a conversational tone is absent (ß = 1.027; *p* < 0.001).

Table 5 adds the tweet source (i.e., government, politician, or public health) to the logistic regression models to illustrate how the tweet source and guiding principle affect tweet engagement. For all models, when each guiding principle is present and the tweet originates from a politician, the odds of receiving an average rate of engagement were over 30 times greater than when the guiding principle is absent and from a government source. Likewise, for all models, when each guiding principle is present and from public health, the odds of an average rate of engagement were over 2 times greater than when the guiding principle is absent and from the government.

### 3.3. Relationship between the Number of Guiding Principles Used and Level of Tweet Engagement

Using two or more guiding principles per tweet increased the odds of receiving an average rate of tweet engagement (Table 6). When two guiding principles were present, the odds of an average tweet engagement rate increased by 1.4 (ß = 0.317; *p* = 0.001); when three guiding principles were present the odds of an average tweet engagement rate increased by 2.0 (ß = 0.705; *p* < 0.001); and when four guiding principles were present the odds of an average tweet engagement rate increased by 2.2 (ß = 0.808; *p* = 0.01). Using zero or one guiding principle in a tweet decreased the odds of receiving an average rate of tweet engagement.

## 4. Discussion

Effective communication during emerging infectious disease can aid key actors including government, politicians, and public health in influencing risk perception and behaviors, thereby reducing the impact of the crisis. Crisis communication serves to inform the public about the risks and recommended health protective behaviors and should include all relevant guiding principles that are known to enhance trust and overall uptake of public health recommendations. Social media platforms, such as Twitter, provide channels that help move actors away from one-way or top-down communication towards interactive communication with the public. Twitter reported its largest gain in daily users globally during COVID-19 [29], making it an important platform by which to evaluate official crisis communication.

### 4.1. Use of Guiding Principles Enhances Engagement

The odds of having average engagement or higher on a tweet were enhanced when clarity, compassion and empathy, conversational tone, and correction of misinformation were present. Higher engagement indicates interaction with the information from an audience that is attentive and responsive to the messaging [33]. Timely and interactive crisis communication is a key aspect of building and maintaining trust in crisis actors [33]. The strategic use of guiding principles has been previously associated with higher engagement during the Ebola [33] and COVID-19 [29] crises. Recent research indicates that a lack of engagement in COVID-19 tweets was associated with actors providing general information that was aimed at the community-level rather than being targeted towards various subpopulations [29].

Call to action and transparency were found to reduce the odds of a tweet having higher than average engagement. The tweets containing a call to action were rarely direct and did not often explain the benefit of visiting the link. A good call to action drives people to the organization’s website or increases the use of resources by using instructive phrases [34]. It outlines the benefit of visiting the link or following the instructions and tells the user where the link will take them [34]. The lack of effective calls to action is likely why this guiding principle was not found to be associated with higher engagement. Similarly, transparency was almost always messages about research funding for COVID-19. While this is an aspect of transparency, messages were missing the most important goal of this guiding principle of sharing balanced information about what is both known and unknown.

### 4.2. Use of Guiding Principles Was Low across All Sources

Our study revealed low and inconsistent use of guiding principles for crisis communication from government, politicians, and public health during COVID-19. A recent study examining COVID-19 tweets also found low use of risk communication strategies across similar account types [29]. Of the 10 accounts we examined, low use of clarity, compassion and empathy, correction of misinformation, and transparency was found. The two most commonly and consistently used guiding principles include conversational tone and call to action, which are best practices for social media communication specifically [22]. However, call to action was often incorporated as a URL at the end of the tweet, without a direct ask or explanation of the link.

The use of the guiding principles contributes to message development and dissemination, which have been linked to various outcomes including increased understanding, reduced uncertainty, maintenance of trust in key actors, alignment of risk perception with actual risk, and increased self-efficacy [5]. As seen in prior research, transparent, clear, compassionate messages are vital to these outcomes [4,5]. On average, transparency was only used in 4.7% of tweets, which is extremely low for such an important guiding principle. A lack of transparency is linked to distrust in key actors, increased belief in disinformation, and contributes to COVID-19 vaccine hesitancy [35]. Once trust is lost, it is extremely difficult to regain [36] and crisis communication will have little persuasive effect [35]. Transparency is the most important strategy for maintaining trust by both the public and official crisis actors [10].

Moreover, customization of health messages is necessary to be significant enough to influence individual behavioral change [37]. A one size fits all approach is not effective for COVID-19 crisis communication, especially when it comes to countering mis- and dis-information [38], overcoming vaccine hesitancy [35], and influencing attitudes and behaviors towards public health measures [39]. The use of guiding principles enhances the personal relevance of crisis messages to the audience by understanding their cultural and personal context, barriers to public health measure uptake, preferred communication channels, and risk perception [5,40].

### 4.3. Source of the Crisis Message Impacts Engagement

Politicians and public health were associated with increased engagement across all guiding principles, with politicians having the greatest influence. Government was also associated with smaller increased odds of higher engagement with all guiding principles except for call to action and transparency. Public health had the highest number of guiding principles found in their tweets, followed by politicians, and then government. Early in the pandemic, a survey found that public health was the most trusted source of COVID-19 information (ranked first by 37%), with government officials ranked fourth (rated first by 6%) [41]. Although public health had the highest number of COVID-19 tweets and incorporated the highest number of guiding principles compared to the other sources, the use of most guiding principles was still inconsistent and low across all sources. The trusted role that public health plays in promoting and protecting health likely influences message perception in a positive manner [3,9,42]. It is interesting that politicians use of guiding principles was highly associated with increased engagement. Although less trusted than public health, higher use of some guiding principles may be increasing the acceptability of their messaging. Conversational tone was highly used by Justin Trudeau and compassion and empathy was highly used by Chrystia Freeland, although on average all politicians had the highest use of both principles. These guiding principles help make the messages more meaningful, build rapport, and acknowledge feelings that are associated with high uncertainty surrounding emerging infectious disease [43].

Public health had less use of compassion and empathy but higher use of clarity and the only instances of correction of misinformation that was found. All guiding principles that were used by public health predicted higher engagement compared to when used by government. Clarity plays an important role in trust through targeting and tailoring messages to increase the relevance, understanding, and uptake of public health measures [44]. Furthermore, during times of high uncertainty, the public feels fear and anxiety, which can lead them to believe in mis- and dis-information and reduce adherence to risk protective measures [29,45,46]. Correction of misinformation plays an important role in addressing the COVID-19 “infodemic” and providing messaging that counters these beliefs from a trusted source [29].

### 4.4. Combination of Guiding Principles Associated with Increased Engagement

Importantly, our research found that an increasing number of guiding principles used per tweet was associated with higher engagement. Tweets that had zero or one guiding principle used decreased the odds of having higher than average engagement. This is unsurprising as tweets that had zero guiding principles are not using any best practices that were found for crisis messages, and thus are not meeting the needs of Twitter users. The tweets that had one guiding principle most often were labelled as call to action, which as previously discussed, were not effectively incorporated into the tweets. These tweets still did not have guiding principles that customized messages to increase understanding and acceptance.

Our previous research examining COVID-19 Facebook posts also found that the inconsistent combination and application of the guiding principles was associated with negative public sentiment [4]. Similarly, research focused on Ebola crisis messages using Instagram and Twitter found that the strategic and consistent application of principles can increase engagement [33]. Finally, research on COVID-19 in Canada with similar actors also found that the use of one or more crisis communication strategies was associated with increased engagement with tweets [29]. The guiding principles are interrelated and likely function together in their application to increase message acceptance and uptake of public health measures, which is of utmost importance during a public health crisis.

### 4.5. Limitations

Our research focused on 10 Twitter accounts representing federal-level government, political, and public health actors. There are many other actors in the system, such as provincial level actors, and across other social media crisis subarenas, such as Instagram, YouTube, etc. The public may engage differently with other types of actors and on other platforms. Further, engagement is measured differently across many studies, making it difficult to make comparisons. We classified engagement as a binary variable (i.e., average or low), but more flexible boundaries leading to high, medium, or low engagement levels could have been used for more detailed analyses. Additionally, although English is the most widely used language on Twitter, COVID-19 related discussions in other languages were not included. Moreover, the keywords that were used to obtain tweets were confined to a limited number of terms, which may have missed desired content. It is also possible that certain demographics are more represented as a result of the actors that were focused on for this research. Our results provide a snapshot of how government, politicians, and public health used guiding principles in their Twitter crisis communication and resulting public engagement. The benefit of this research is the relatively large dataset across several sources and a significant period of time during COVID-19. Actors can use this information to better design and customize their crisis messages for increased engagement and acceptance.

Future research would benefit from analyzing the sentiment of replies to tweets and comparing how sentiment relates to engagement. The use of the Premium Twitter API allowed for collection of replies but did not attach them to the original tweet, limiting our analysis potential.

## 5. Conclusions

We found low and inconsistent use of guiding principles for COVID-19 tweets from government, politicians, and public health. Our study shows the combination of guiding principles can improve tweet engagement. When tweets included conversational tone or compassion and empathy specifically, the odds of higher engagement increased. Additionally, we found increased odds of tweet engagement as the number of guiding principles that were used per tweet increased. Lastly, beyond just the guiding principles, it is important to consider the source of crisis communication. In this study, across all guiding principles, politicians and public health had higher odds of greater tweet engagement than tweets from government. Overall, this research demonstrates how trusted actors can better incorporate guiding principles to increase engagement with their crisis messages.

## Figures and Tables

**Table 1:** Key features of guiding principles for crisis communication using social media.

Guiding Principle	Key Features	Example Tweet
Call to Action ^a^	Asks the public to do something as a result of the information [1,6,22] e.g., Visit a website, share the post, watch a video, look at infographic, help others, etc.	Do your part and download it today: URL link.
Clarity	Uses plain language (i.e., common terms, parallel form, short sentences) [23,24,25] Conveys complex information visually [22] Targets and tailors information to audience(s) [22,24,26]	The Government of Canada is working hard to provide all Canadians from coast to coast to coast with access to #COVID19 vaccines. Learn more about what makes a vaccine safe.
Compassion and Empathy	Validates and shows emotion [24] Expresses concern and willingness to impact future tragedy [1,24,26]	A few weeks ago, my six-year-old son asked me: Dad, is COVID-19 forever? It’s not. And we need to keep that in mind. Because yes, this sucks—but better days are coming. If we keep working hard and following public health guidelines, we will get through this together.
Conversational Tone ^a^	Balances friendly conversational tone with professionalism [22,27] Uses first or second person, contractions, and implements good spelling and grammar [22]	We’ve reached 5 million downloads of the #COVIDAlert app! By using the app, we can help protect ourselves, our loved ones, and our communities from #COVID19. Do your part and download it today.
Correction of Misinformation	Addresses and corrects misinformation including rumors and myths [3,7,23,25,26]	Federally designated quarantine sites, typically hotel rooms, are not internment camps. #Misinformation is circulating that Canada is using concentration camps for #COVID19 quarantine. This is completely false.
Transparency	Provides honest and accurate information [6,23,25] Shares strengths and weakness, uncertainties, and completeness of information [24,25,26] Communicates future research/decisions/how they will go about finding answer [7,23]	Today, the Government of Canada released projections on #COVID19. Our actions now can determine what our country will look like in the weeks and months to come.

^a^ Social media best practice. Adapted from MacKay, Colangeli, Gillis, et al., 2021.

**Table 2:** Twitter account name, Twitter handle, number of followers as of 9 August 2021, and the number of tweets that were collected.

Twitter Account Name	Twitter Handle	Number of Followers	Number of Tweets Collected
Government			
Canada	@Canada	980,535	17
CIHR	@CIHR_IRSC	61,127	185
Finance Canada	@FinanceCanada	87,346	310
Politicians			
CanadianPM	@CanadianPM	543,830	411
Chrystia Freeland	@cafreeland	229,273	65
Erin O’Toole	@erinotoole	134,125	66
Justin Trudeau	@JustinTrudeau	5,694,063	453
Public Health			
Cdn Public Health Assoc.	@CPHA_ACSP	11,476	109
Dr. Theresa Tam	@CPHO_Canada	274,920	3219
Health Canada and PHAC	@GovCanHealth	390,621	1218

**Table 3:** Guiding principles that were used in tweets from government, politician, and public health Twitter accounts.

	Call to Action *n* (%)	Clarity *n* (%)	Compassion and Empathy *n* (%)	Conversational Tone *n* (%)	Correction of Misinformation *n* (%)	Transparency *n* (%)
**Government**	299 (97.08%)	28 (9.09%)	35 (11.36%)	95 (30.84%)	0 (0.00%)	27 (8.77%)
Canada	11 (100.00%)	5 (45.45%)	2 (18.18%)	7 (63.64%)	0 (0.00%)	4 (36.36%)
CIHR	108 (97.30%)	11 (9.91%)	9 (8.11%)	18 (16.22%)	0 (0.00%)	22 (19.82%)
Finance Canada	180 (96.77%)	12 (6.45%)	24 (12.90%)	70 (37.63%)	0 (0.00%)	1 (0.54%)
**Politicians**	524 (87.77%)	56 (9.38%)	144 (24.12%)	367 (61.47%)	0 (0.00%)	18 (3.02%)
CanadianPM	241 (97.57%)	21 (8.50%)	43 (17.41%)	63 (25.51%)	0 (0.00%)	12 (4.86%)
Chrystia Freeland	28 (71.79%)	1 (2.56%)	24 (61.54%)	26 (66.67%)	0 (0.00%)	1 (2.56%)
Erin O’Toole	38 (97.44%)	0 (0.00%)	13 (33.33%)	18 (46.15%)	0 (0.00%)	0 (0.00%)
Justin Trudeau	217 (79.78%)	34 (12.50%)	64 (23.53%)	260 (95.59%)	0 (0.00%)	5 (1.84%)
**Public Health**	1797 (65.87%)	399 (14.63%)	279 (10.23%)	1388 (50.88%)	12 (0.44%)	65 (2.38%)
Cdn Public Health Assoc.	52 (80.00%)	11 (16.92%)	2 (3.08%)	18 (27.69%)	0 (0.00%)	1 (1.54%)
Dr. Theresa Tam	1035 (53.60%)	25 (1.29%)	228 (11.81%)	860 (44.54%)	4 (0.21%)	47 (2.43%)
Health Canada and PHAC	710 (96.99%)	363 (49.59%)	49 (6.69%)	510 (69.67%)	8 (1.09%)	17 (2.32%)

**Table 4:** Results from six univariate logistic regression analyses each with a different guiding principle as the predictor variable for the level of tweet engagement.

	Odds Ratio (exp(ß)) ^a^	Estimate (ß)	Standard Error	z Value	*p* Value	*AUC*
(Intercept)	0.185	−1.689	0.087	−19.499	<0.001	
Call to Action	0.898	−0.107	0.103	−1.041	0.30	0.511
(Intercept)	0.165	−1.800	0.051	−35.244	<0.001	
Clarity	1.273	0.241	0.130	1.851	0.06	0.514
(Intercept)	0.152	−1.886	0.052	−35.940	<0.001	
Compassion and Empathy	2.160	0.770	0.120	6.396	<0.001	0.551
(Intercept)	0.093	−2.380	0.085	−27.980	<0.001	
Conversational Tone	2.794	1.027	0.103	10.000	<0.001	0.621
(Intercept)	0.171	−1.766	0.047	−37.525	<0.001	
Correction of Misinformation	1.169	0.156	0.776	0.201	0.84	0.500
(Intercept)	0.172	−1.760	0.048	−36.972	<0.001	
Transparency	0.848	−0.165	0.290	−0.569	0.57	0.502

^a^ Absence of the guiding principle is the referent category for each logistic regression model.

**Table 5:** Results from six multivariate logistic regression analyses, each with a different guiding principle and tweet source (i.e., government, politician, public health) as predictor variables for the level of tweet engagement.

	Odds Ratio (exp(ß)) ^a^	Estimate (ß)	Standard Error	z Value	*p* Value	*AUC*
(Intercept)	0.064	−2.747	0.359	−7.651	<0.001	0.792
Call to Action	0.454	−0.789	0.127	−6.198	<0.001	
Politicians	35.014	3.556	0.349	10.204	<0.001	
Public Health	2.025	0.706	0.351	2.012	0.04	
(Intercept)	0.028	−3.569	0.339	−10.522	<0.001	0.775
Clarity	1.764	0.568	0.149	3.799	<0.001	
Politicians	37.650	3.628	0.349	10.412	<0.001	
Public Health	2.632	0.968	0.346	2.795	0.005	
(Intercept)	0.029	−3.544	0.339	−10.453	<0.001	0.770
Compassion and Empathy	1.367	0.312	0.143	2.191	0.03	
Politicians	35.864	3.580	0.348	10.274	<0.001	
Public Health	2.740	1.008	0.346	2.913	0.004	
(Intercept)	0.020	−3.899	0.345	−11.309	<0.001	0.785
Conversational Tone	2.611	0.960	0.116	8.311	<0.001	
Politicians	30.636	3.422	0.350	9.779	<0.001	
Public Health	2.262	0.816	0.348	2.347	0.02	
(Intercept)	0.030	−3.503	0.338	−10.355	<0.001	0.764
Correction of Misinformation	2.450	0.896	0.778	1.152	0.25	
Politicians	37.110	3.614	0.348	10.382	<0.001	
Public Health	2.712	0.998	0.346	2.884	0.004	
(Intercept)	0.030	−3.492	0.339	−10.292	<0.001	0.763
Transparency	0.876	−0.132	0.334	−0.395	0.69	
Politicians	36.853	3.607	0.349	10.351	<0.001	
Public Health	2.706	0.996	0.347	2.873	0.004	

^a^ Absence of the guiding principle and government source are the referent categories.

**Table 6:** Results from univariate logistic regression analyses with the number of guiding principles used as the predictor variable for the level of tweet engagement.

	Odds Ratio (exp(ß)) ^a^	Estimate (ß)	Standard Error	z Value	*p* Value	*AUC*
(Intercept)	0.177	−1.729	0.049	−34.940	<0.001	
Zero	0.719	−0.330	0.158	−2.090	0.04	0.516
(Intercept)	0.217	−1.528	0.056	−27.252	<0.001	
One	0.504	−0.686	0.104	−6.586	<0.001	0.577
(Intercept)	0.153	−1.875	0.059	−31.612	<0.001	
Two	1.373	0.317	0.097	3.253	0.001	0.536
(Intercept)	0.152	−1.886	0.053	−35.639	<0.001	
Three	2.024	0.705	0.117	6.027	<0.001	0.550
(Intercept)	0.169	−1.780	0.048	−37.433	<0.001	
Four	2.243	0.808	0.317	2.546	0.01	0.507

^a^ Having any number of guiding principles other than what is listed is the referent category for each logistic regression model.

## Data Availability

Data are available upon request from the corresponding author Melissa MacKay at melissam@uoguelph.ca.
